# Comparative Analysis of Digestion Methods for Bile Proteomics: The Key to Unlocking Biliary Biomarker Potential

**DOI:** 10.1021/acs.analchem.4c01766

**Published:** 2024-08-26

**Authors:** Adam M. Thorne, Martijn Hoekzema, Robert J. Porte, Folkert Kuipers, Vincent E. de Meijer, Justina C. Wolters

**Affiliations:** † Department of Liver Transplantation and HPB Surgery, University of Groningen and University Medical Center, 9713 GZ Groningen, The Netherlands; ‡ UMCG Comprehensive Transplant Center, 9700 RB Groningen, The Netherlands; § Department of Applied Life Sciences, Institute for Life Science and Technology, Hanze University Groningen, 9747 AS Groningen, The Netherlands; ∥ Erasmus MC Transplant Institute, Department of Surgery, Division of HPB and Transplant Surgery, University Medical Center Rotterdam, 3015 GD Rotterdam, The Netherlands; ⊥ European Research Institute for the Biology of Ageing (ERIBA), University of Groningen and University Medical Center Groningen, 9713 AV Groningen, The Netherlands; # Department of Pediatrics, University of Groningen and University Medical Center Groningen, 9700 RB Groningen, The Netherlands

## Abstract

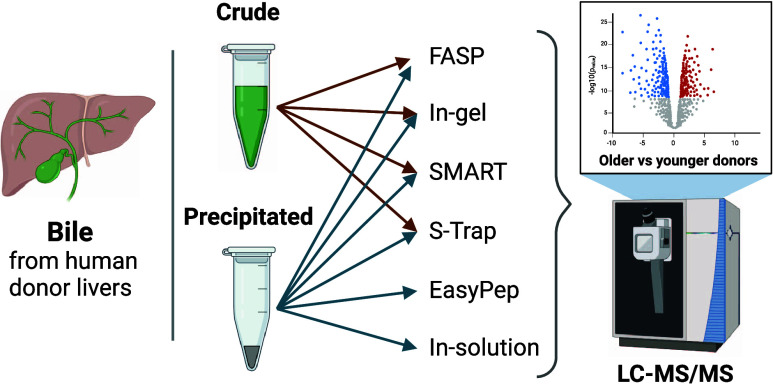

Background: Bile’s potential to reflect the health of the biliary system has led to increased attention, with proteomic analysis offering deeper understanding of biliary diseases and potential biomarkers. With the emergence of normothermic machine perfusion (NMP), bile can be easily collected and analyzed. However, the composition of bile can make the application of proteomics challenging. This study systematically evaluated various trypsin digestion methods to optimize proteomics of bile from human NMP livers. Methods: Bile was collected from 12 human donor livers that were accepted for transplantation after the NMP viability assessment. We performed tryptic digestion using six different methods: in-gel, in-solution, S-Trap, SMART, EasyPep, and filter-aided sample purification, with or without additional precipitation before digestion. Proteins were analyzed using untargeted proteomics. Methods were assessed for total protein IDs, variation, and protein characteristics to determine the most optimal method. Results: Methods involving precipitation surpassed crude methods in protein identifications (4500 vs 3815) except for in-gel digestion. Filtered data (40%) resulted in 3192 versus 2469 for precipitated and crude methods, respectively. We found minimal differences in mass, cellular components, or hydrophobicity of proteins between methods. Intermethod variability was notably diverse, with in-gel, in-solution, and EasyPep outperforming others. Age-related biological comparisons revealed upregulation of metabolic-related processes in younger donors and immune response and cell cycle-related processes in older donors. Conclusions: Variability between methods emphasizes the importance of cross-validation across multiple analytical approaches to ensure robust analysis. We recommend the in-gel crude method for its simplicity and efficiency, avoiding additional precipitation steps. Sample processing speed, cost, cleanliness, and reproducibility should be considered when a digestion method is selected for bile proteomics.

## Introduction

Liver transplantation is the preferred treatment option for patients with end-stage liver disease, certain metabolic diseases, and selected malignancies. The evolution of liver transplantation, marked by significant improvements in surgical technique, logistics, and postoperative care, has resulted in increasing numbers of liver transplants that have been crucial in providing life-saving care. Despite this, there remains a shortage of donor livers considered suitable for transplantation, and up to 20% of patients die while waiting for a transplant.^1^ This shortage has led to a greater reliance on marginal livers from suboptimal donors, including grafts from donation after circulatory death (DCD) donors, donors with advanced age, and those with extensive comorbidities, in an effort to address high mortality. However, these suboptimal grafts carry a higher risk of post-transplant complications and, despite rising numbers of deceased organ donors, many of these donated livers are considered too high risk for transplantation and are subsequently discarded.^2^

In this regard, advancements in organ preservation methods have emerged as a key area of focus. Among these, normothermic machine perfusion (NMP) has gained popularity in recent years as a method for dynamic preservation and assessment of human donor liver quality prior to transplantation.^3,4^ This platform makes it possible to maintain donor livers at near-physiological conditions outside the body, sustaining cellular metabolism and function. This allows for accurate assessment of functional processes.^5^ Consequently, high-risk organs previously deemed unsuitable for transplantation may now be reconsidered. Current viability assessment strategies vary, reflecting the complexity of decision making in organ transplantation based on point-of-care biochemical information alone.^3,6^ At our center, cholangiocellular criteria, assessed via bile, appear vital in determining the viability of the biliary tree and avoidance of post-transplant cholangiopathies in initially discarded high-risk donor livers.^3,7,8^

NMP not only allows for preservation and assessment of donor livers for transplantation but also presents a unique opportunity to better study liver function through evaluation of the processes involved in bile production. Bile is a complex fluid that plays an essential role in the digestion and excretion of endogenous and exogenous waste products and the intestinal absorption of dietary fats and sterols. Bile is produced by hepatocytes in a process mainly driven by active (energy-dependent) transport of bile acids into the bile canaliculi that drain into the intrahepatic and, finally, the extrahepatic bile ducts. The direct proximity of bile components to the cells that constitute the biliary tree suggests that bile can represent a potential indicator to evaluate the overall health of the biliary system. Detailed assessment of bile composition and the profiling of its proteomic content during NMP may yield insights into liver physiology, pathology, and the effectiveness of the preservation process.^9^

Despite the importance of bile in determining organ viability, it remains relatively understudied compared to other, more accessible sample types such as plasma, urine, and tissue. This, in part, can be attributed to the challenges associated with bile collection, with traditionally invasive techniques such as endoscopic retrograde cholangiopancreatography, cholecystectomy, or nasobiliary drain among the more common ways to collect samples. These procedures are inherently associated with higher risk and potential for complications when compared with a simple blood collection and are therefore not routinely performed solely for research purposes. Importantly, with NMP, bile becomes a noninvasive, easily collectible sample type, providing a novel, longitudinal means to assist clinical decision making for machine-perfused livers. In recent years, clinical proteomics has leveraged advanced technologies to identify potential biomarkers and molecular pathways that can indicate liver and biliary disease states. Biliary diseases, including cholestasis and primary sclerosing cholangitis, can be categorized by changes in bile composition.^10−13^ The application of clinical proteomics to bile analysis during NMP is particularly promising and could lead to the discovery of novel biomarkers that are specific to liver viability and function during perfusion.^14,15^ Biomarker discovery can be further extended to other perfusion techniques, for example, normothermic regional perfusion (NRP) or post-transplant monitoring of bile through transanastomic bile T-drains. However, the complex composition of bile, high in lipids, salts, and detergents, as well as a host of other organic and anorganic molecules, complicates protein enrichment and analysis by mass spectrometry (MS), with high variation in protocols used for proteomics analysis.

In this study, we aimed to systematically evaluate a variety of readily available trypsin digestion methods for proteomics analysis of bile, focusing on samples obtained from transplanted clinical NMP livers. Methods were evaluated on total protein IDs, reproducibility, variability, protein characteristics, and the potential effect of these factors on biological comparison. We aim to identify methods that yield high-quality proteomic data, thereby maximizing the potential of bile as a valuable source of biomarkers. The findings of our study will provide insights into the optimization of bile proteomics workflows, which could enhance our understanding of the molecular mechanisms underlying various hepatocellular and biliary diseases.

## Experimental Section

### Machine Perfusion and Sample Collection

Bile was collected from nationally declined human donor livers that underwent NMP viability testing. NMPs were carried out as previously described using a Liver Assist device (XVIVO, Groningen, The Netherlands).^3^ Following a period of static cold storage during transportation from the donor hospital to the transplant center, donor livers first underwent at least 1 h of dual hypothermic oxygenated machine perfusion (DHOPE, 8–12 °C), followed by 1 h of controlled oxygenated rewarming (COR, 20–37 °C), and subsequent NMP (37 °C) for 2.5 h. After 2.5 h NMP, liver viability was assessed according to previously established criteria.^3^ Livers deemed viable were maintained on the NMP device at 37 °C during the recipient hepatectomy and transplanted. Livers deemed nonviable were rejected for transplantation, the perfusion terminated, and the liver discarded. For this study, the bile samples used were taken at time of viability assessment (2.5 h after start of NMP), from transplanted livers only. For biological comparison, bile samples from “older” (>69 years old) and “younger” (<60 years old) donors were used. Donor data were extracted from the EuroTransplant database. All relevant ethical regulations were adhered to.

### Protein Quantitation

**Figure 1:**
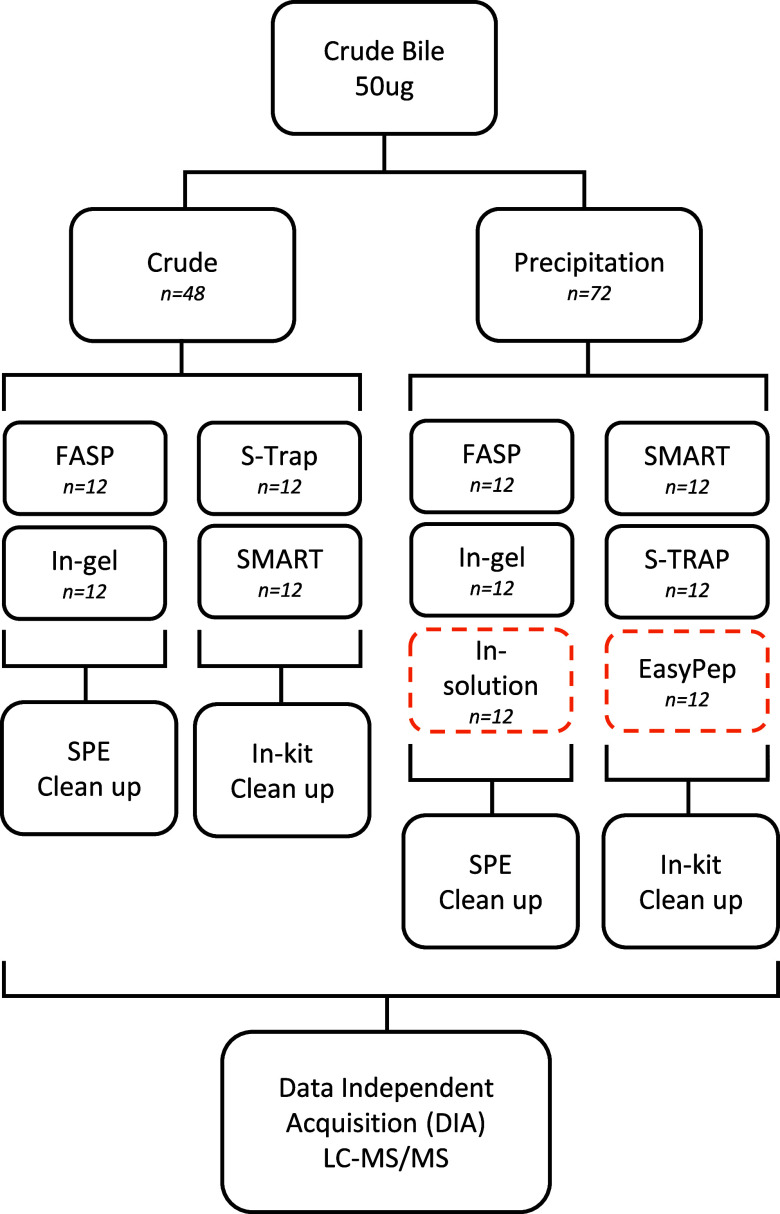
Schematic overview of sample preparation steps. All methods were compared with and without an additional methanol/chloroform precipitation step, except in-solution and EasyPep, which include precipitation steps in their protocols (highlighted with orange dashed lines). In solution, in-gel and FASP digestions (crude and precipitated) underwent C18 SPE cleanup, whereas EasyPep, SMART, and S-Trap digestions (crude and precipitated) had a cleanup step in the kit protocols. After cleanup, all samples were analyzed using DIA LC-MS/MS. Abbreviations: FASP; filter-aided sample purification, S-Trap; suspension trapping, DIA; data-independent acquisition, LC-MS/MS; liquid chromatography–tandem mass spectrometry, SPE; solid-phase extraction.

For initial protein quantitation, 100 μL of bile was centrifuged at 15,000*g* for 15 min to remove debris and distributed across all methods in both crude and precipitated arms. Protein concentrations were determined using a micro-BCA assay (Pierce, Thermo Fisher Scientific, Waltham, MA) according to the manufacturer’s instructions. Bile volumes containing 50 μg protein were taken (0.8–3.0 μL) forward for digestion by each of the described methods (Figure 1).

### Protein Precipitation

Protein precipitation using methanol/chloroform was used as a comparison to crude bile to assess removal of contaminants and potential losses in digestion methods that do not already include a precipitation step (FASP, in-gel, S-trap, and SMART). Bile volumes containing 50 μg of protein were diluted to 200 μL using 100 mM ammonium bicarbonate (ABC). 600 μL of methanol and 150 μL of chloroform were added to the diluted sample and vortexed for 30 s, after which 450 μL of Milli-Q water was added. Samples were then vortexed again for 30 s and centrifuged at 14,000*g* for 1 min. The resulting aqueous phase was removed and discarded, and 450 μL of methanol was added. Samples were vortexed and centrifuged at 14,000*g* for 1 min, and the supernatant was discarded. The resulting protein pellets were air-dried and resuspended in 50 μL of 100 mM ABC where applicable.

### EasyPep (Mini) Digestion

Bile samples were processed according to the manufacturer’s instructions (Thermo Fisher Scientific, MA). 50 μg of (methanol/chloroform) precipitated protein was resuspended in 100 μL of kit “Lysis Buffer”. 50 μL of “Reduction Solution” and 50 μL of “Alkylation Solution” were added to the sample and incubated at 95 °C for 10 min. After reduction/alkylation, samples were cooled to room temperature, before adding trypsin at a ratio of 1:100. Samples were vortexed for 30 s and incubated at 37 °C for 3 h. 50 μL of “Digestion Stop Solution” was added after 3 h to quench the reaction. For cleanup, the kit Peptide Clean-up columns were used. Columns were centrifuged at 3000*g* for 2 min to remove the storage liquid. The protein digest was then transferred to the columns and centrifuged at 1500*g* for 2 min. Next, columns were washed with 300 μL of “Wash Solution A”, followed by two rounds of 300 μL of “Wash Solution B”. Columns were centrifuged at 1500*g* for 2 min after each wash step, and the flow-through discarded. Finally, 300 μL of “Elution Solution” was added to each column and the effluent was collected. Cleaned peptides were then dried using a vacuum concentrator and stored at −80 °C until use.

### Filter-Aided Sample Purification Digestion

For filter-aided sample purification (FASP) digestion, Amicon Ultra 0.5 mL 30 kDa molecular weight filters were used (Merk-Millipore, Darmstadt, Germany). Filters were equilibrated with 200 μL of 8 M urea and centrifuged at 14,000*g* for 20 min. Volumes containing 50 μg of protein were added to the filters. Samples were reduced by adding 100 μL of 20 mM dithiothreitol in 8 M urea for 30 min at room temperature, after which they were centrifuged at 14,000*g* for 10 min. Samples were then alkylated using 100 mM iodoacetamide (IAA) for 30 min in the dark at room temperature, followed by the same centrifuge step. Buffer exchange was then performed using 2 × 100 μL of 8 M urea and then 3 × 100 μL of 50 mM ABC, with centrifuging at 14,000*g* for 10 min in between each step. Trypsin was then added at a trypsin-protein ratio of 1:100 and incubated at 37 °C overnight. Filters were then centrifuged at 5000*g* for 1 min upside down to collect the peptides, followed by a wash of 100 μL of 0.5 M NaCl. Finally, a second wash of 200 μL of Milli-Q water centrifuged at 14,000*g* for 10 min was performed to collect any residual peptides. The resulting solution containing peptides was acidified using 10 μL of 100% formic acid (FA) and taken forward for solid-phase extraction (SPE) cleanup.

### In-Gel Digestion

50 μg of protein, from crude or precipitated sample, was combined with 6.25 μL 4× loading buffer (Abcam, Cambridge, United Kingdom) and topped up to 25 μL using 100 mM ABC. Samples were heated to 70 °C for 10 min, cooled, and added to a gel (4–12% Bis-Tris plus, Thermo Fisher Scientific, Waltham, MA) and run at 100 V for 5 min. The gels were then stained for 1 h using Coomassie Blue stain (InstantBlue, Abcam, Cambridge, United Kingdom), and destained in Milli-Q water overnight. The protein-containing gel bands were then cut from the gel and further destained/dehydrated using 300 μL steps of 30% acetonitrile (ACN) in 100 mM ABC, 50% ACN in 100 mM ABC, and 100% ACN before being air-dried. The proteins were reduced and alkylated using 10 mM dithiothreitol at 57 °C for 30 min and 55 mM IAA in the dark at room temperature for 30 min, respectively. The gel pieces were then dehydrated using 300 μL of 100% ACN and dried. The proteins were then digested using sequencing-grade trypsin (Promega, Wisconsin) at a trypsin-to-protein ratio of 1:100 and left to digest at 37 °C overnight. The digested peptides were extracted from the gel pieces using 60 μL of 5% formic acid, 75% ACN, and taken forward for SPE cleanup.

### In-Solution Digestion

Volumes containing 50 μg protein were diluted to 200 μL using 100 mM ABC. Reduction and alkylation of proteins were carried out as described in the in-gel methods. Proteins were then precipitated using the methanol/chloroform protocol, as described. The resulting pellet was resuspended in 50 μL of 8 M urea by vortexing and sonication. The final urea concentration was diluted with 250 μL of Milli-Q water to give <1 M urea when trypsin was added. Trypsin was added at a ratio of 1:100 and digested at 37 °C overnight. The resulting peptides were then taken forward for SPE cleanup.

### S-Trap Digestion

S-Trap digestion was performed using S-Trap micro-sample preparation kits (Protifi, New York) according to the manufacturer’s instructions. 50 μg of protein were diluted to 11.5 μL using 100 mM ABC. 11.5 μL of buffer 1 (2× lysis buffer; 10% sodium dodecyl sulfate (SDS), 100 mM triethylammonium bicarbonate (TEAB), pH 8.5) was added, and samples were vortexed and sonicated. Samples were then reduced by adding 1 μL of 120 mM tris(2-carboxyethyl)phosphine (TCEP) and incubating at 55 °C for 15 min. Alkylation was followed by adding 1 μL of 500 mM methylmethanethiosulfonate (MMTS) in isopropanol and incubated at room temperature for 10 min. Samples were then acidified using 2.5 μL of 12% phosphoric acid. 150 μL of 90% methanol/100 mM TEAB was added to the samples and mixed before adding to the S-Trap columns. Columns with the sample were then centrifuged at 10,000*g* for 30 s, after which 400 μL of 90% methanol/100 mM TEAB was added and repeated three times. Trypsin was added at a ratio of 1:100 and diluted to a final volume of 20 μL using 50 mM TEAB and incubated overnight at 37 °C. Samples were then eluted using a stepwise approach with 40 μL of 50 mM TEAB, 0.2% FA, and 50% ACN in water, respectively. Collected peptides were then dried in a vacuum concentrator and resuspended in 0.1% FA for mass spectrometry (MS) analysis.

### SMART Digestion

SMART digest (Thermo Fisher Scientific, Waltham, MA) was performed according to the manufacturer’s instructions. 50 μg of protein was diluted to 50 μL using 100 mM ABC. 150 μL of “Digest Buffer” was then added and the samples were vortexed for 30 s. Samples were then added to the SMART digest tubes and placed on a heat block at 70 °C with shaking at 1400 rpm for 3 h. Samples were then cooled to room temperature before spinning at 1000*g* for 2 min to separate the beads from the sample. Cleanup was achieved using the kits’ SOLAμ SPE plates. SPE columns were conditioned using 200 μL of ACN. Columns were then equilibrated with 200 μL of 0.1% trifluoracetic acid (TFA). Samples were diluted 1:1 with 0.1% TFA and added to the columns, with care taken to run the samples through the column slowly. The columns were then washed with 500 μL of 0.1% TFA, before eluting the cleaned peptides with 2 × 25 μL of 75% ACN in Milli-Q water. The peptides were then dried using a vacuum concentrator and taken forward for MS analysis.

### Solid-Phase Extraction (SPE) Cleanup

SPE columns (GracePure 1 mL C-18-Aq column, Thermo Fisher Scientific, Waltham, MA) were conditioned twice with 1 mL 100% ACN/0.1% FA and equilibrated twice with 0.1% FA. Samples were then mixed 1:1 with 0.1% FA and applied to the column. Bound peptides were then washed twice with 1 mL of 0.1% FA, and eluted with two rounds of 400 μL of 50% ACN/0.1% FA. Samples were then dried in a vacuum concentrator and resuspended in 0.1% FA for MS analysis.

### Mass Spectrometry

Liquid chromatography (LC)-MS/MS analysis was performed using an Orbitrap Exploris 480 quadrupole-Orbitrap hybrid mass spectrometer equipped with a nanoelectrospray ion source (Thermo Fisher Scientific, Waltham, MA), coupled with a front-end high-field asymmetric waveform ion mobility (FAIMS) device (Thermo Fisher Scientific, Waltham, MA). Chromatographic separation of peptides was performed by liquid chromatography (LC) on a nano-HPLC system (Ultimate 3000, Dionex, Thermo Fisher Scientific, Waltham, MA), using a nano-LC column (Acclaim PepMapC100 C18, 75 μm × 50 cm, 2 μm, 100 Å, Dionex, buffer A: 0.1% v/v formic acid in Milli-Q-H_2_O, buffer B: 0.1% v/v formic acid in acetonitrile). 6 μL of sample (1 μg/μL) was loaded into a trap column (μPrecolumn cartridge, Acclaim PepMap100 C18, 5 μm, 100 Å, 300 μm × 5 mm, Dionex) using buffer A as a transport liquid. Peptides were separated on the nano-LC column using a linear gradient from 2 to 45% buffer B over 75 min at a flow rate of 300 nL/min, followed by a column wash using 80% buffer B. The mass spectrometer was operated in positive ion mode and data-independent acquisition mode (DIA) using isolation windows of 12 *m*/*z* with a precursor mass range of 300–1200. There was no window overlap used, and window placement optimization was “on”. The orbitrap resolution was set to 120,000 and DIA scan resolution at 30,000. Two compensation voltages (−60 and −45 V) were used with the FAIMS device. Samples were randomized before sample preparation; however, sample order remained consistent with this randomization order across all methods, including injection for LC-MS/MS analysis.

### Data Analysis and Statistics

MS raw data was searched using Spectronaut software (version 15, Biognosys) using library-free DIA analysis workflows. Oxidation and deamidation were set as variable modifications, and carbamidomethylation was set as a fixed modification. Data was searched against human protein sequences using the UPR_homoSapiens_FASTA file (20,401 entries) Downloaded from www.uniprot.org (December 12, 2022). MS1 values were used for protein quantitation and normalized using global normalization. Quantitative values for identified protein groups were processed using Perseus software (v1.6.6.4). Protein IDs were filtered to include proteins present in at least 40% of samples within each method. Heatmaps were generated in R using log 2 transformed values converted to *z*-score. For biological comparison, volcano plots from parametric Student *t* test (assuming normal distribution of data) with a permutation-based FDR of 0.05 to assess multiple comparisons was employed. *t* test calculations were performed in Perseus using log 2 transformed values with missing values replaced using imputation based on normal distribution of values. Visualization of volcano plots was performed using R. Biological pathway analysis was performed in CytoScape (version 3.9.1, National Resource for Network Biology) software using STRING protein databases (https://string-db.org/). Receiver operator characteristic analysis was performed using SPSS 29 (IBM) using age groups (“older” and “younger” donors) as binary classifiers. The Table of Contents was created with BioRender.com. Raw MS data used for proteomics have been deposited to the ProteomeXchange Consortium via the PRIDE partner repository with the data set identifier PXD051062.^16^ All other data are available in the main text or Supporting Information.

## Results

### Donor Characteristics

Donor characteristics of the livers from which bile was collected for this study are presented in Table 1. For later biological comparison, samples were stratified by age: younger donors (<60 years old, *n* = 6) and older donors (>69 years old, *n* = 6). The median donor age of all donors was 64 (interquartile range (IQR) 52–71), with the median ages of the younger and older donor groups being 52 (IQR 48–56) and 72 (IQR 70–73; *p* = 0.002), respectively. All livers included in this study were from initially discarded DCD donors that were transplanted following fulfillment of previously established viability criteria.^3^

**Table 1:** Donor Characteristics for Included Livers^a^

	all (*n* = 12)		younger (*n* = 6)		older (*n* = 6)		*p*-value
donor characteristics							
age (years)	64	(52–71)	52	(48–56)	72	(70–73)	**0.002**
body mass index (kg/m^2^)	26	(25–31)	28	(24–34)	26	(25–27)	0.790
gender							1.000
male	9	(75%)	5	(83%)	4	(67%)	
female	3	(25%)	1	(17%)	2	(33%)	
cause of death							0.913
trauma	4	(33%)	2	(33%)	2	(33%)	
CVA	3	(25%)	1	(17%)	2	(33%)	
anoxia	4	(33%)	3	(50%)	1	(17%)	
other	1	(8%)	0	(0%)	1	(17%)	
donor type							1.00
DCD	12	(100%)	6	(100%)	6	(100%)	
DBD	0	(0%)	0	(0%)	0	(0%)	
time from withdrawal of life support to circulatory arrest (min)	17	(15–34)	36	(15–59)	16	(15–19)	0.457
time from circulatory arrest to cold perfusion (min)	16	(15–18)	17	(15–17)	16	(15–18)	0.900
functional donor warm ischemia time^b^ (min)	29	(25–49)	47	(28–69)	27	(22–32)	0.305
last sodium (mmol/L)	144	(140–145)	142	(139–144)	145	(143–146)	0.413
last AST (U/L)	37	(26–139)	118	(54–162)	26	(21–33)	0.065
last ALT (U/L)	41	(23–119)	129	(66–159)	27	(20–32)	**0.041**
last GGT (U/L)	45	(20–93)	83	(35–140)	32	(17–45)	0.132
last ALP (U/L)	85	(63–110)	79	(65–138)	85	(55–101)	0.589
hepatectomy time (min)	36	(32–40)	38	(33–40)	35	(32–39)	0.957
static cold ischemia time (min)	248	(183–274)	244	(200–268)	248	(186–271)	0.937
DRI^c^	2.60	(2.48–2.93)	2.47	(1.97–2.53)	2.99	(2.72–3.19)	**0.009**

a Continuous data are presented as median (IQR), categorical data as a number (percentage).

b Time from donor saturation <80% or mean arterial pressure <60 mm/Hg to initiation of in situ cold flushing in the donor. Static cold ischemia time was defined as the time between initiation of cold flushing in the donor and start of DHOPE. Abbreviations: ALP; alkaline phosphatase, ALT; alanine aminotransferase, AST; aspartate aminotransferase, CVA; cerebrovascular accident, DBD; donation after brain death, DCD; donation after circulatory death, DRI; donor risk index, GGT; γ glutamyl transferase.

c Validated scoring tool to assess the risk of liver graft failure.

### Protein Identifications

To assess potential differences in coverage of the bile proteome, we compared six different digestion methods (Figure 1). The composition of bile (high concentrations of detergent bile acids, lipids, and salts) can negatively influence protein enrichment, digestion, and MS analysis. Because of this, we compared crude bile samples with samples that had undergone protein precipitation prior to digestion (with the exception of in-solution and EasyPep methods that already include precipitation steps in the protocol).

**Figure 2:**
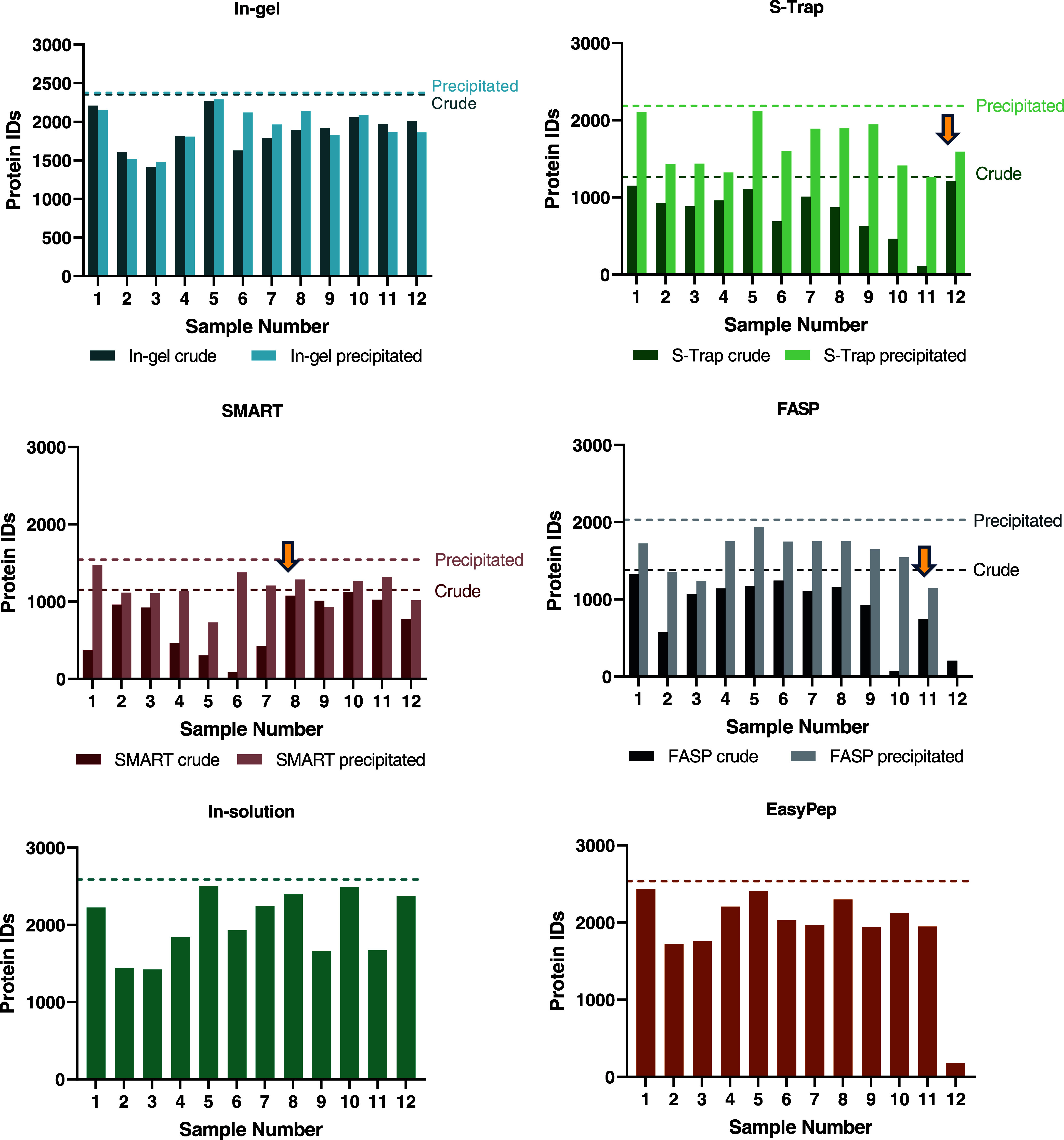
Total number of unique proteins identified in each digestion method. The total number of proteins identified in each sample are represented by the bars (for both crude and precipitated samples). The total number of proteins identified within each method group (e.g., in-gel crude, in-gel precipitated) are represented by dashed lines on the *y*-axis, where the color of the line represents the method. Samples run after FAIMS cleaning are indicated with arrows.

In total, 4650 unique proteins were identified across all digestion methods (crude and precipitated conditions inclusive). To reduce noise and perform analysis on only the most robustly identifiable proteins, we applied a filter to the identified proteins. The filter cutoff was defined using the lowest median percentage presence of proteins in all samples across all methods. The total median protein coverage for each method can be seen in Table 2. While the median coverage varied considerably between methods (ranging from 41.7 to 66.7%), a cutoff of 40% was implemented for all methods, based on the lowest observed coverage (41.7%; SMART crude and S-Trap crude), resulting in a protein having to be present in at least 5 of the 12 samples to be retained for analysis. This approach was used to maintain consistency in the results and, furthermore, ensured that proteins uniquely present in one biological condition were retained post filtering. The number of proteins before and after filtering for each method can be seen in Table 2. S-Trap crude was most affected by filtering, with 48.7% of proteins being removed. The method with the fewest proteins removed was in-gel precipitation with 27.5% of proteins being removed. In methods using crude samples, a total of 2469 unique proteins were identified. In contrast, methods using precipitated samples identified a total of 3192 unique proteins. While this can partially be attributed to a larger number of methods using precipitation (*n* = 6 precipitated vs *n* = 4 crude), total protein identifications (IDs) remained higher in precipitated methods that have crude counterparts (FASP, in-gel, SMART, and S-Trap) with a total of unique 2807 proteins, accounting for an increase in protein IDs of 13.6%. An exception to this was in-gel digestion, in which only a minimal difference was observed. Total protein IDs for each sample and each method can be seen in Figure 2.

**Table 2:** Protein IDs and Filtering^a^

method	total IDs	median protein coverage	total IDs (40% filtered)	number of proteins filtered	percentage proteins filtered	median CV (%) post filter
EasyPep precipitated	3524	66.7	2537	987	28	84.5
FASP crude	2243	50	1381	862	38.4	154.1
FASP precipitated	3103	58.3	2030	1073	34.6	73.8
in solution precipitated	3688	66.7	2589	1099	29.8	82.1
in-gel crude	3405	66.7	2356	1049	30.8	72.2
in-gel precipitated	3276	66.7	2375	901	27.5	69.7
SMART crude	2236	41.7	1152	1084	48.5	110
SMART precipitated	2243	58.3	1544	699	31.2	78.4
S-Trap crude	2464	41.7	1265	1199	48.7	140.3
S-Trap precipitated	3194	58.3	2186	1008	31.6	92.2
crude methods (total)	3815		2469	1346	35.2	
precipitated methods (total)	4500		3192	1308	29.1	

a Median protein coverage represents the median percentage of samples in which a given protein was present. A value of 40% indicates that the median presence of any given protein occurs in at least 40% of the samples. A filtering percentage of 40% median protein coverage, accounting for the lowest observed across all methods, was applied to all methods.

To assess variation and overlap in identified proteins between different methods, we conducted a comparative analysis of all protein IDs within crude (Figure S1A) and precipitated (Figure S1B) conditions. For methods using crude samples, 851 proteins were present in all methods, comprising 35% of the total proteins detected. Notably, the in-gel crude digestion methods demonstrated superior performance relative to that of other crude methods. This was evident from both the highest number of protein IDs (2356) and the highest number of proteins unique to the method (736). Furthermore, in-gel crude digestion had the highest number of overlapping proteins with all other crude methods, encompassing over 95% of proteins identified in all crude techniques. SMART crude digest identified the fewest proteins and had the least overlap with all other crude methods, although it was not substantially less so than S-Trap crude or FASP crude. For methods using precipitated samples, 1272 proteins were common between all methods, again accounting for 40% of the total protein IDs (Figure S1B). In-solution precipitation had both the highest number of total proteins identified and the most unique proteins of any precipitated method. However, EasyPep and in-gel precipitation methods were comparable. SMART precipitation remained the poorest for protein IDs, identifying the fewest total protein IDs (notably fewer than any other precipitated method) and the least overlap with other methods. S-Trap precipitation and FASP precipitation performed similarly. Protein crossover between crude and precipitated conditions of each method is depicted in Figure S1C. Comparing the crude and precipitated conditions within methods, in-gel demonstrated the highest overlap, with more than 85% of proteins identified present in both methods. S-Trap and FASP methods benefited most from precipitation, showing 72.8 and 46.9% increases in protein IDs, respectively.

### Variation between Methods

**Figure 3:**
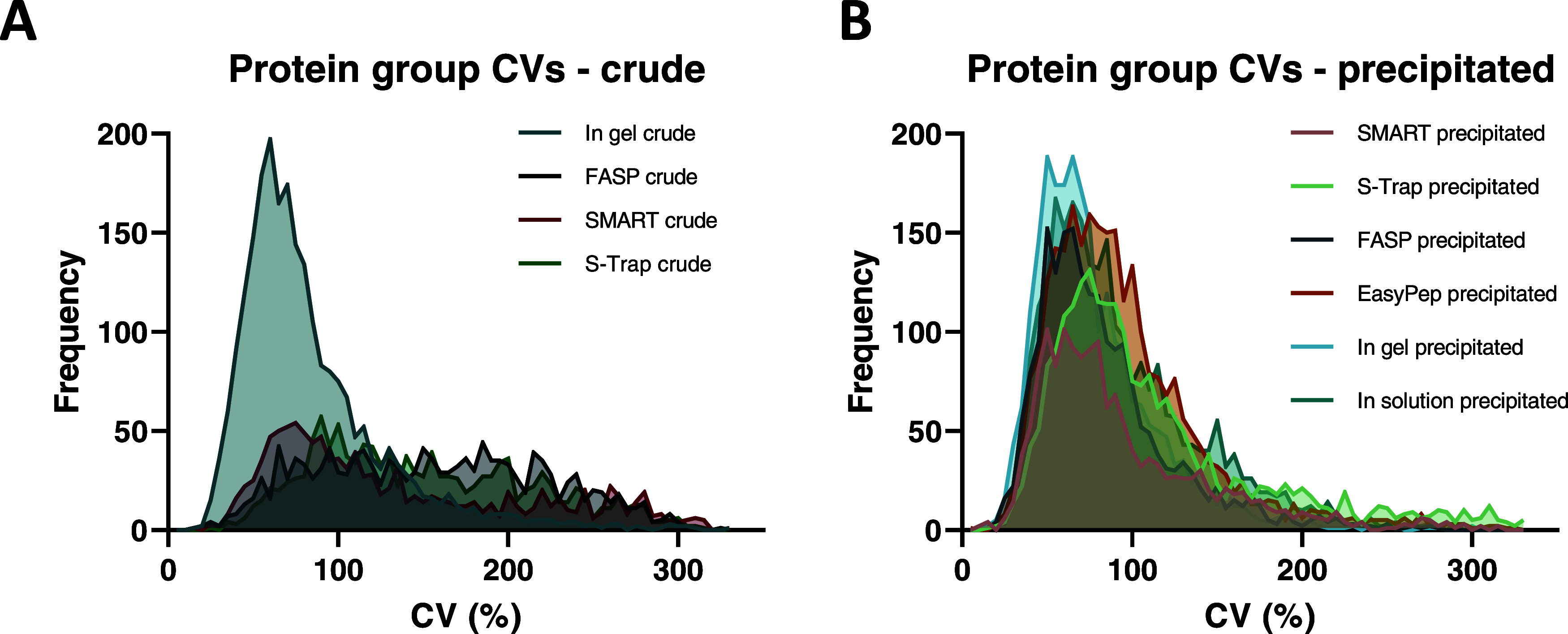
Histograms of coefficient of variation (CV) distribution. (A) CV distribution at protein group level in crude digestion methods. (B) CV distribution at protein group level in precipitated digestion methods.

To assess the variation between methods and conditions, we analyzed the coefficients of variation (CV). At the protein group level, crude methods (Figure 3A) showed substantially larger degrees of variation compared to methods using precipitation (Figure 3B). Interestingly, the in-gel crude showed a substantially lower median CV and a higher frequency of lower CVs, a pattern comparable to that seen in precipitated methods. CV values in precipitation methods were largely similar. Details of CV values and protein IDs can be seen in Table 2. Individual CV histograms for each method at the protein group level can be seen in Figure S2. Taken together, methods with a precipitation step showed a higher number of total protein IDs and lower variation, with in-gel crude digestion being the exception. A further source of potential variation between methods is the presence of proteins reflecting contamination such as skin keratins. To investigate if more “hands-on” methods, such as in-gel digestion, contained higher levels of potential contaminants, we compared protein profiles of all methods against a list of known contaminant proteins. Of the 20 human contaminant proteins investigated, 6 were present in at least one method. We observed no substantial difference in protein intensity between the two methods. However, this does not discount the potential for contamination in more labor-intensive methods (Figure S3).

**Figure 4:**
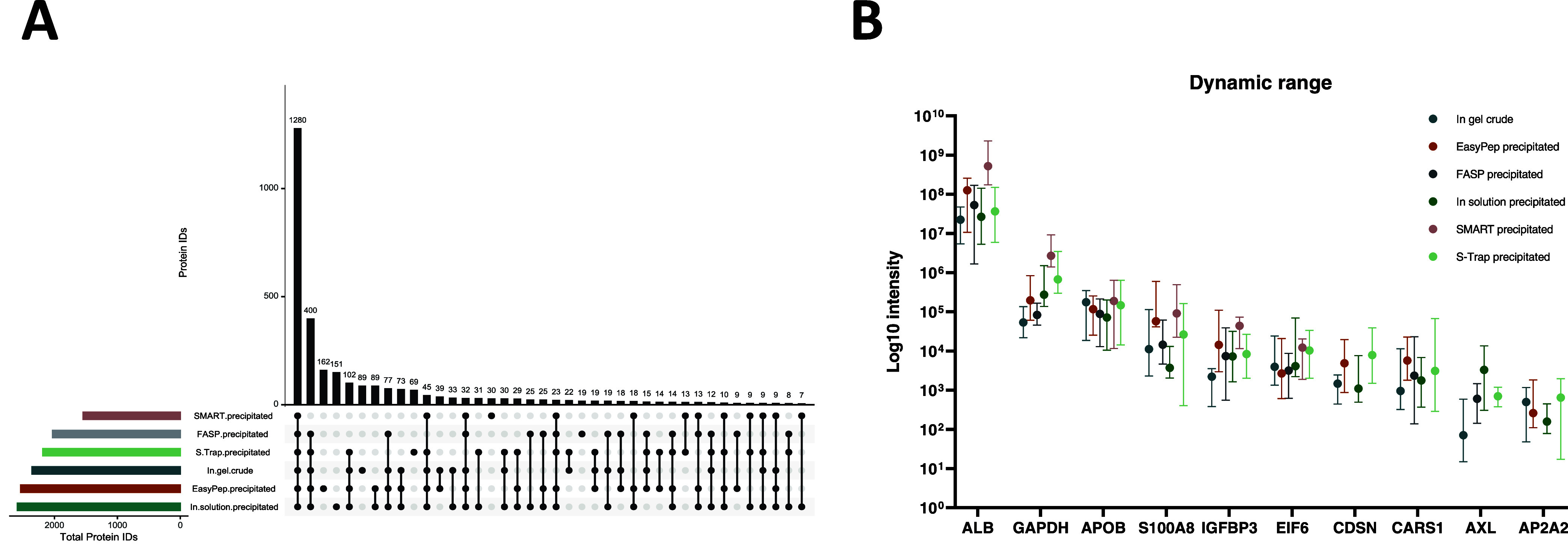
Comparative analysis of protein crossover and intensity. (A) UpSet plot for the top 6 methods selected for further comparison. Total number of protein identifications are represented by the lower left bars. Bars in the upper part of the plot show the number of proteins present in different method combinations. (B) Dynamic range plot showing intensity (median with interquartile range) for 10 selected proteins ranging from high to low intensity.

With these results, we selected the best-performing conditions from each method for further analysis. As in-gel crude and precipitated conditions were comparable, the crude method was taken forward, as the extra step of precipitation did not give superior results, requires more time, and can introduce the potential for protein losses or contamination. This resulted in in-gel crude, in-solution precipitated, EasyPep precipitated, FASP precipitated, and S-Trap precipitated being selected for further comparison. The corresponding plot of total protein ID overlap between the top 6 methods can be seen in Figure 4A.

### Protein Characteristics

We next assessed if any of the top 6 selected methods preferentially enriched specific protein subsets by analyzing cellular components, grand average of hydropathy (GRAVY) index, and protein mass. We observed no significant differences between any of the methods in the percentage frequency or type of cellular components present. SMART precipitated digest appeared most variable to other methods, showing a slightly higher frequency of cytoplasm, blood microparticle, and actin cytoskeleton-related proteins, and a lower frequency of mitochondrial inner membrane-related proteins (Figure S4A). GRAVY index also showed very little difference between methods. Median and interquartile range (IQR) GRAVY index scores were highly similar between methods. In gel, crude digest appeared to have a more balanced range of hydropathy score, with a shorter negative-side tail than other methods (Figure S4B). Similarly, all methods showed a comparable pattern in protein mass, again with the only discernible difference being due to total protein IDs (Figure S4C).

While the characteristics of identified proteins remained consistent across all methods, we observed notable differences in their detected intensities. Dynamic range plots for all methods (Figure S5) appeared relatively similar, again with the main difference being the number of total proteins identified. The exception to this is the SMART digest, where there appears to be a loss of proteins in the lower intensity range. Using a subset of 10 proteins selected for their varying intensity and function (ALB, AP2A2, APOB, AXL, CARS1, CD8N, EIF8, GAPDH, IGFBP3, and S100A8), we saw that despite similar dynamic range profiles, intensities of individual proteins varied across methods (Figure 4B). Fluctuating intensities between methods increased with lower-intensity proteins. However, with SMART precipitated digest, we observed considerably higher intensities of ALB and GAPDH compared with other methods. This may be a result of lower total protein IDs, particularly at lower intensities, which places a higher relative abundance on more intense proteins. Despite variations in intensity between methods, GAPDH, a traditional housekeeping target for quantitative assays, appeared to have consistent and close IQRs within all methods (Figure 4B). Of the selected proteins, APOB demonstrated the least variation between methods, highlighting the importance of selecting stable and robustly quantifiable potential housekeeping targets (Figure 4B).

Taken together, protein digest methods do not show bias for identifying any particular subset of proteins. However, the intensities of proteins may vary substantially between methods, which may have implications for differential quantitative analysis of biological variables.

### Biological Comparison of Older and Younger Donor Livers

**Figure 5:**
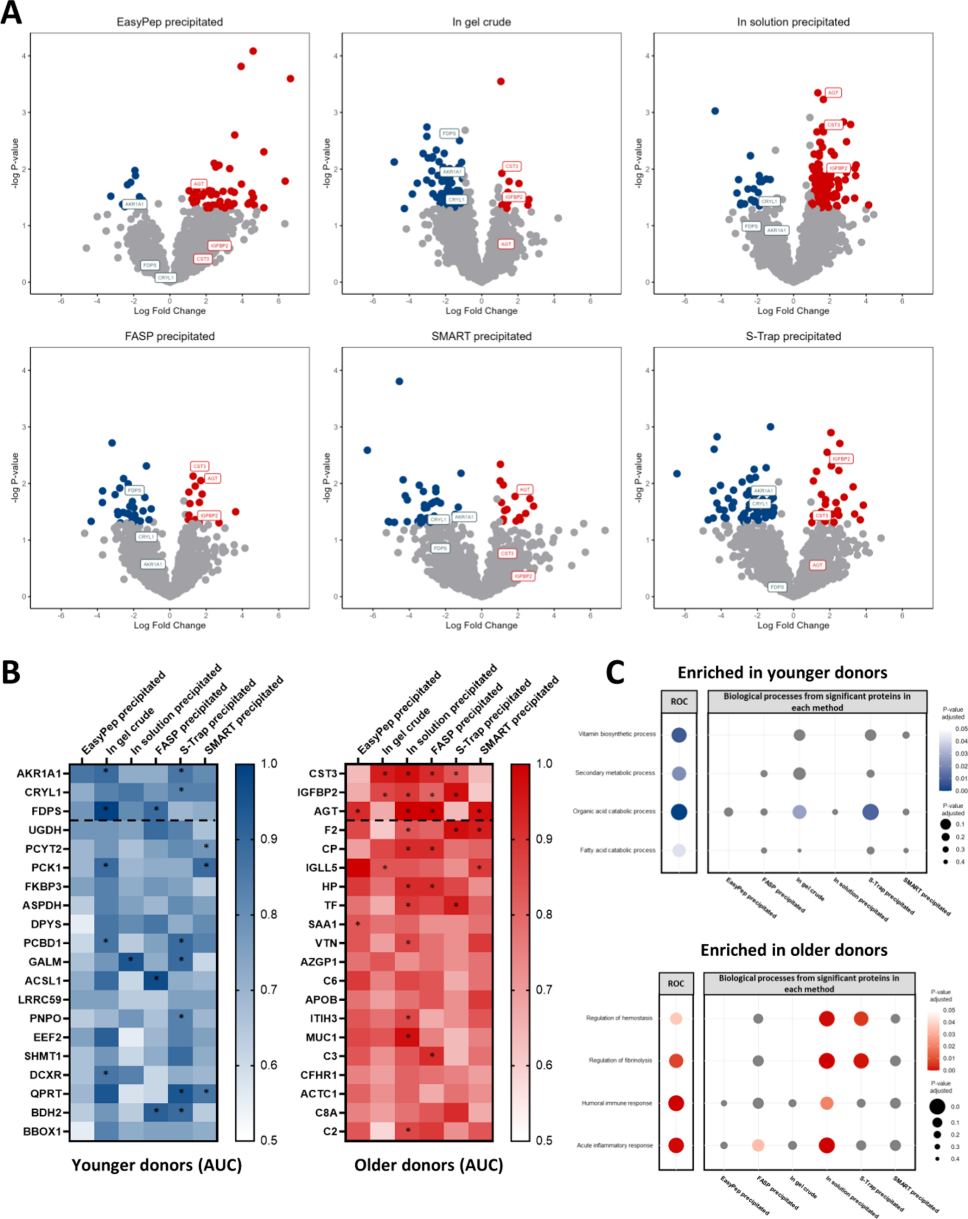
Biological comparison between older and younger donors. (A) Volcano plots showing differential abundance of older versus younger donors in the top 6 selected methods. Significant proteins (*p* < 0.05, >2-fold change) are highlighted in red for proteins enriched in older donors, and in blue for proteins enriched in younger donors. Labeled proteins represent three high area under the curve (AUC) proteins shared between all methods. (B) Heatmaps showing proteins with high AUC in younger (blue) and older (red) donors within all top 6 methods. Significant proteins from differential analysis in volcano plots are indicated with an asterisk. The three proteins at the top of each heatmap are shown in each volcano plot in panel (A). (C) Dot plots showing Gene Ontology (GO) biological processes in younger and older donors. Color gradients represent adjusted *p*-value of significant biological processes. Gray is used for processes where involved proteins are present, but the process did not reach significance. The size of each point represents adjusted *p*-value in significant and nonsignificant biological processes. Receiver operator curve (ROC) section is reflective of processes involving the high AUC proteins in panel (B). Section containing methods is reflective of processes involving differentially significant proteins displayed in the volcano plots in panel (A).

To assess the applicability of the tested methods in biological comparisons, we performed differential analysis for age-related effects, comparing bile proteomes from older (*n* = 6) and younger (*n* = 6) donor livers. We observed a moderate number of differentially abundant proteins across all methods (Figure 5A), with in-solution and EasyPep weighted more toward proteins enriched in older donors. Conversely, in-gel crude digest showed a slight bias toward proteins enriched in younger donors. However, all methods appeared to capture a reasonably balanced representation of biological differences between both groups. Direct comparison of significantly different proteins was limited as a result of differences in total protein IDs (some differentially abundant proteins in one method may be proteins not identified in another method) and variation in protein intensities demonstrated by dynamic range. This variation also hindered direct comparisons of pathway enrichment between methods.

To overcome this, we performed a receiver operator curve (ROC) analysis on all proteins identified in each method and ranked them based on a combination of area under the curve (AUC), presence of the protein in each method, and standard deviation across methods. This approach was used to identify common proteins among all methods and to assess the association of each protein with the given biological condition (younger or older donors). From this analysis, the top 20 proteins were subjected to Gene Ontology (GO) biological process analysis to identify any common pathways present in younger and older donors across all selected methods. Although the top 20 proteins generally had strong associations with the biological condition, not all proteins were considered significantly differentially abundant (*p*-value <0.05, >2-fold-change) (Figure 5B). To demonstrate differential abundance variability between methods, the top 3 identified proteins from the sorted ROC analysis in younger (AKR1A1, CRYL1, and FDPS) and older (CST3, IGFBP2, and AGT) donors are labeled on each of the methods’ volcano plots. While there is considerable variation in some cases, all methods show significant differential abundance (or close to) of 1 or more of these top 3 proteins, suggesting a degree of similarity between all methods.

To investigate age effects further, we plotted the significant GO biological processes identified from these ROC proteins (Figure 5C). This revealed significant enrichment of metabolic-related processes in younger donors, and immune response and cell cycle-related processes in older donors. Aware that these analyses were biased toward proteins commonly associated with all methods, we then performed GO biological process analysis on proteins with significant differential abundance (*p*-value <0.05, >2-fold-change) in each method. We then applied the results to the biological processes observed in the ROC analysis. From this, we saw that the majority of processes from the ROC analysis in younger and older donors were also significant or at least present in the significantly differentially abundant proteins of each method (Figure 5C). Additionally, extended biological process plots (Figure S6) show consistent enrichment of metabolic processes in younger donors and immune response and cell cycle-related processes in older donors in all methods. Taken together, despite variation in the number of protein IDs and differential abundance of identified proteins, there remain consistent, overarching biological themes between all methods.

## Discussion

Proteomic analysis of bile from human livers undergoing ex situ machine perfusion presents a unique opportunity for a better understanding of the molecular pathogenesis of biliary complications and potential biomarker discovery for objective viability assessment. Despite its potential, the scarcity of large-scale proteomic studies underscores the inherent difficulties associated with sample acquisition and the uniquely challenging chemical composition of bile for LC-MS/MS analysis. In this study, we evaluated six different protein digestion methods on bile samples taken from initially discarded human donor livers considered suitable for transplantation after reassessment using NMP. We found that protein IDs varied considerably between methods, although there were no specific protein subsets preferentially enriched or lost. Furthermore, we observed enrichment of specific biological processes in comparisons of bile from younger versus older donors.

There are a range of techniques available for protein preparation in human sample types, with the simplest approaches involving direct protein digestion without steps to remove or reduce potential contaminants. While there is no current standardized sample preparation protocol, the composition of bile, with high abundances of potentially interfering compounds of lipids, detergents, and salts, has resulted in the majority of present studies using some form of protein precipitation/purification prior to digestion. These include top-14 affinity columns (MARS; Agilent, CA),^17^ lipid depletion using Cleanascite (Ligo-Chem, Fairfield, NJ),^11,17,18^ and/or a combination of organic solvents.^10−12,19−22^ Our approach was to employ traditional methanol/chloroform precipitation, chosen for its accessibility (avoiding the use of expensive columns or chemicals) and for minimizing additional sample preparation steps that could result in protein losses. From our results, we found that although proteomic analysis on crude bile is possible, the case remains that a method of protein enrichment prior to digestion, either a form of protein precipitation or purification (e.g., running through a gel) is essential to prevent instrument contamination and sample variation. In many of the methods using crude bile, we observed a sharp and continuous decline in protein IDs not reflected in precipitated/gel methods, suggesting substantial cumulative contamination during LC-MS/MS analysis (Figure 2). Cleaning of the MS, particularly the FAIMS device, was required much more frequently in crude sample methods (excluding in the gel crude digest). Despite substantial increase in protein IDs after cleaning, it was not feasible to clean after each sample run, furthermore highlighting the impracticality of crude digest methods for analysis of larger cohorts necessary in clinical proteomics studies. While the FAIMS device may result in higher sensitivity to contaminations (resulting in a failed run and stopping the sample queue), these were limited to the front end and prevented contaminants from entering the instrument. Thus, the frequent need for cleaning and sensitivity to contamination underscores the limitations of methods using crude bile for large-scale applications.

The comparison of various protein digestion methods revealed various advantages and disadvantages. After protein precipitation or purification, total protein IDs were largely comparable across all methods, with the exception of the SMART digest that notably underperformed. Solution-based methods, specifically, in-solution digest and EasyPep, alongside in-gel crude digest, were most effective for total protein IDs. Despite variations in methodology, no clear differentiation was observed in enriched protein characteristics such as cellular components, mass, and hydrophobicity. This suggests that the complexity associated with proteomics of bile cannot solely be attributed to the digestion method. This is further illustrated by similarities in the protein mass range between gel- and solution-based methods. Previous work comparing in-gel to in-solution protein digestion in kidney machine perfusion perfusate found a broader range of protein masses present in in-solution digestion, particularly at higher mass ranges;^23^ a difference not observed in our results. This may be attributed to the lower complexity of perfusate compared to bile, fewer protein IDs in perfusate, and liver being a more metabolically active organ than kidney.

As differences in protein characteristics appear to be minimal, other considerations may be more influential in method selection. Solution-based methods are largely recognized for the speed and simplicity of sample preparation, using fewer steps than alternative approaches, such as gel digests. However, without prior protein precipitation, solution-based methods may introduce larger variation, inconsistent protein IDs, and contamination. Although Kit-based methods such as EasyPep, S-Trap, and SMART offer simplicity, they introduce additional costs and may not provide proportional benefits. Conversely, in-gel digestion, while relatively labor-intensive and potentially leading to greater protein losses due to increased handling, did not translate to fewer protein IDs compared to precipitated, solution-based methods as expected.^24^ Moreover, in-gel digestion allows for manual fractionation of protein bands to decrease sample complexity, improving proteome depth, and offers the option to manually deplete high-abundance proteins (such as albumin) by excising the protein-containing band.^25,26^ This can result in better quantitation of low-abundance proteins, particularly in data-dependent acquisition MS modes.^26^ However, band excision for removing unwanted proteins (such as albumin) may result in co-depletion of unrelated proteins due to the low resolution of SDS-polyacrylamide gel electrophoresis (PAGE). While this approach may not be appropriate for studies using large sample numbers, sample fractionation is an effective way of improving proteome depth or targeting specific protein ranges.^11,17,26,27^ Nonetheless, labor-intensive strategies are balanced with reproducibility and removal of potential contaminants, which may avoid delays for instrument cleaning and sample reruns.

The choice of the protein digestion method may also be influenced by the biological questions to be addressed. In-gel digestion has previously been investigated for its propensity to identify membrane-associated proteins more completely through preparation steps using SDS, although this was not evident in our results.^28^ This may be a result of the sample time point used in this study. Membrane-associated proteins are typically expected in bile at earlier time points during NMP (at the highest levels in the earliest bile samples available; around 30 min) due to the cellular damage incurred during the donor procedure and subsequent cold storage.^29^ Our previous work showed a significant flush out of cellular debris proteins at early time points, which rapidly decrease during the perfusion.^9^ Moreover, the bile samples analyzed in this study were derived from livers that were transplanted after the viability assessment, which typically have lower levels of cellular injury in the bile ducts. These observations highlight that the adaptive capacity of the method to align with study design, throughput, specific biological questions, and reproducibility, particularly across samples with changing composition, is a significant factor to consider.

Despite variations in protein profiles, overarching biological processes were observed between young donors and old donors in all methods. Direct comparison of methods was problematic due to differences in protein IDs and intensities. Differential analysis of protein profiles across selected methods revealed varying degrees of similarity in significantly changed proteins, but common themes emerged at both protein and biological process level. Younger donors showed an association with increased metabolic-related processes. This is likely reflective of a reduction in hepatobiliary metabolic activity that occurs with increasing age.^30,31^ In contrast, bile from livers from older donors showed an increase in the number of proteins associated with immune responses and cell cycle-related processes. Furthermore, processes involved in collagen regulation and glycoprotein metabolic processes have been shown to be upregulated in the effects of hepatocyte function as a result of age-dependent changes.^32^ The upregulation of cell cycle processes, including JAK-STAT pathways, blood vessel remodeling, and extracellular matrix organization suggest a response to cellular damage; traditionally a concern with older organ donors due to increased vulnerability to cellular injury, particularly in bile ducts.^33^ Furthermore, single-cell RNA sequencing of liver immune cells has shown proliferative properties, suggesting protective/regenerative effects of a heightened immune response in viable livers in response to cellular damage.^9,34^ Despite this, all samples included in this study were from viable transplanted livers. We have previously shown that regenerative processes within the bile duct are key to liver viability.^9,35^ This suggests that molecular processes rather than age might be a more appropriate measure for the acceptance of high-risk donor livers.

In conclusion, although there are a variety of protein digestion methods applicable to bile, our study shows that the method used can influence the results obtained. A method of protein purification, either through chemical precipitation or gel filtering, is essential for reduced variability, to improve consistency of protein identification, and to reduce potential for contamination. We found no significant differences in the characteristics of identified proteins between methods; however, technical boundaries of digestion methods should be considered and defined by experimental choices. The variations in the dynamic range observed across methods can significantly influence biomarker discovery, necessitating caution when selecting differential proteins as potential biomarkers if one relies solely on a single method. This emphasizes the importance of cross-validation across multiple analytical approaches to ensure the robustness of the potential biomarkers. Flexibility and adaptation to existing protocols for application to specific characteristics of sample types should be carefully considered to increase the potential of proteome analysis.

## Supporting Information Available

Additional figures containing extra information about the comparison of the digestion methods (Figures S1–S5) and the biological comparison between younger and older donors (Figure S6) (PDF)
