# Supplementary material for: Comparative Analysis of Digestion Methods for Bile Proteomics: The Key to Unlocking Biliary Biomarker Potential
Source: Anal Chem. 2024 Aug 26;96(36):14393–404. doi: 10.1021/acs.analchem.4c01766 (PMC11391409; doi:10.1021/acs.analchem.4c01766)
Supplement: Supplementary file 1 — ac4c01766_si_001.pdf [file ac4c01766_si_001.pdf]

Supporting Information

Comparative Analysis of Digestion Methods for Bile Proteomics: The Key to Unlocking Biliary Biomarker Potential

Adam M. Thorne, [†, ‡] *Martijn Hoekzema ,* [†,§] *Robert J. Porte ,* [†,∥] *Folkert Kuipers ,* [⊥,#] *Vincent E. de Meijer,* [†, ‡,▿] *and Justina C. Wolters* [*,#,▿]

[†] Department of Liver Transplantation and HPB Surgery, University of Groningen, and University Medical Center, 9713 GZ Groningen, The Netherlands

[‡] UMCG Comprehensive Transplant Center, 9700 RB Groningen, The Netherlands

[§] Department of Applied Life Sciences, Institute for Life Science and Technology, Hanze University Groningen, 9747 AS Groningen, The Netherlands

[∥] Erasmus MC Transplant Institute, Department of Surgery, Division of HPB and Transplant Surgery, University Medical Center Rotterdam, 3015 GD Rotterdam, The Netherlands

[⊥] European Research Institute for the Biology of Ageing (ERIBA), University of Groningen, and University Medical Center Groningen, 9713 AV Groningen, The Netherlands

[#] Department of Pediatrics, University of Groningen, and University Medical Center Groningen, 9700 RB Groningen, The Netherlands

[*] Corresponding author: j.c.wolters@umcg.nl
[▿] V.E.d.M. and J.C.W. shared senior authorship

Table of contents

Supplementary figure 1. Overlap of total unique proteins identified in each digestion method.

Supplementary figure 2. Histograms of coefficient of variation (CV) distribution.

Supplementary figure 3. Presence of potential contaminant proteins.

Supplementary figure 4. Protein characteristics in the top 6 selected methods.

Supplementary figure 5. Protein intensity dynamic ranges in the top 6 selected methods.

Supplementary figure 6. Biological comparison between younger and older donors.

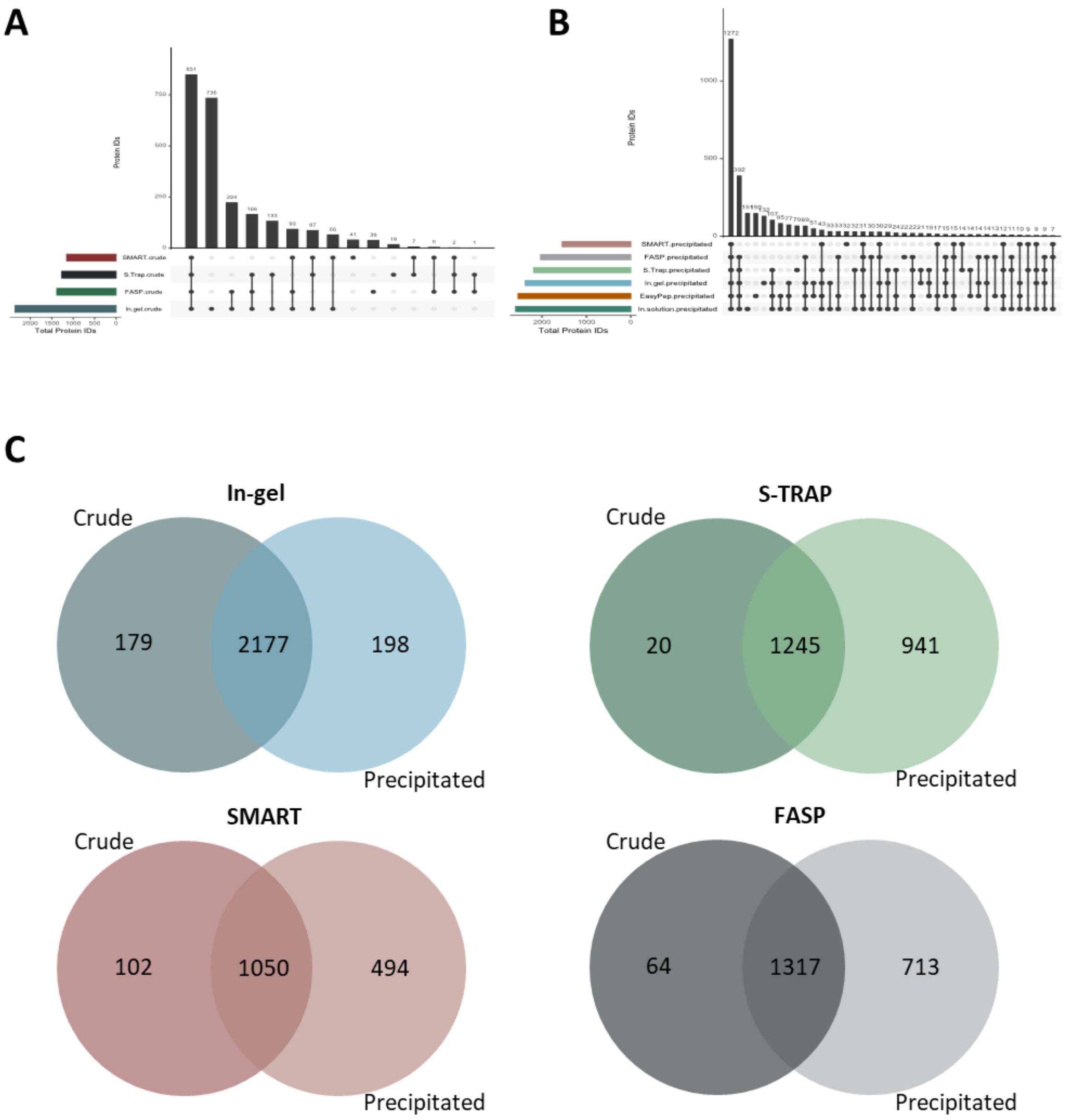

Supplementary figure 1. Overlap of total unique proteins identified in each digestion method. (A) UpSet plot showing protein crossover in crude methods only. (B) UpSet plot showing protein crossover in precipitated methods only. Total number of protein identifications are represented by the lower left bars. Bars in the upper part of the plot show the number of proteins present in different method combinations. (C) Protein identification crossover between crude and precipitated conditions of each method.

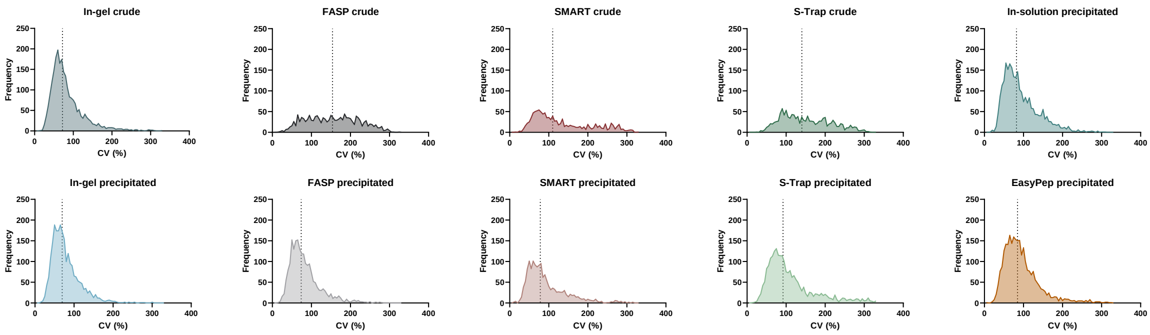

Supplementary figure 2. Histograms of coefficient of variation (CV) distribution. CV distribution at protein group level for individual crude and precipitated digestions methods.

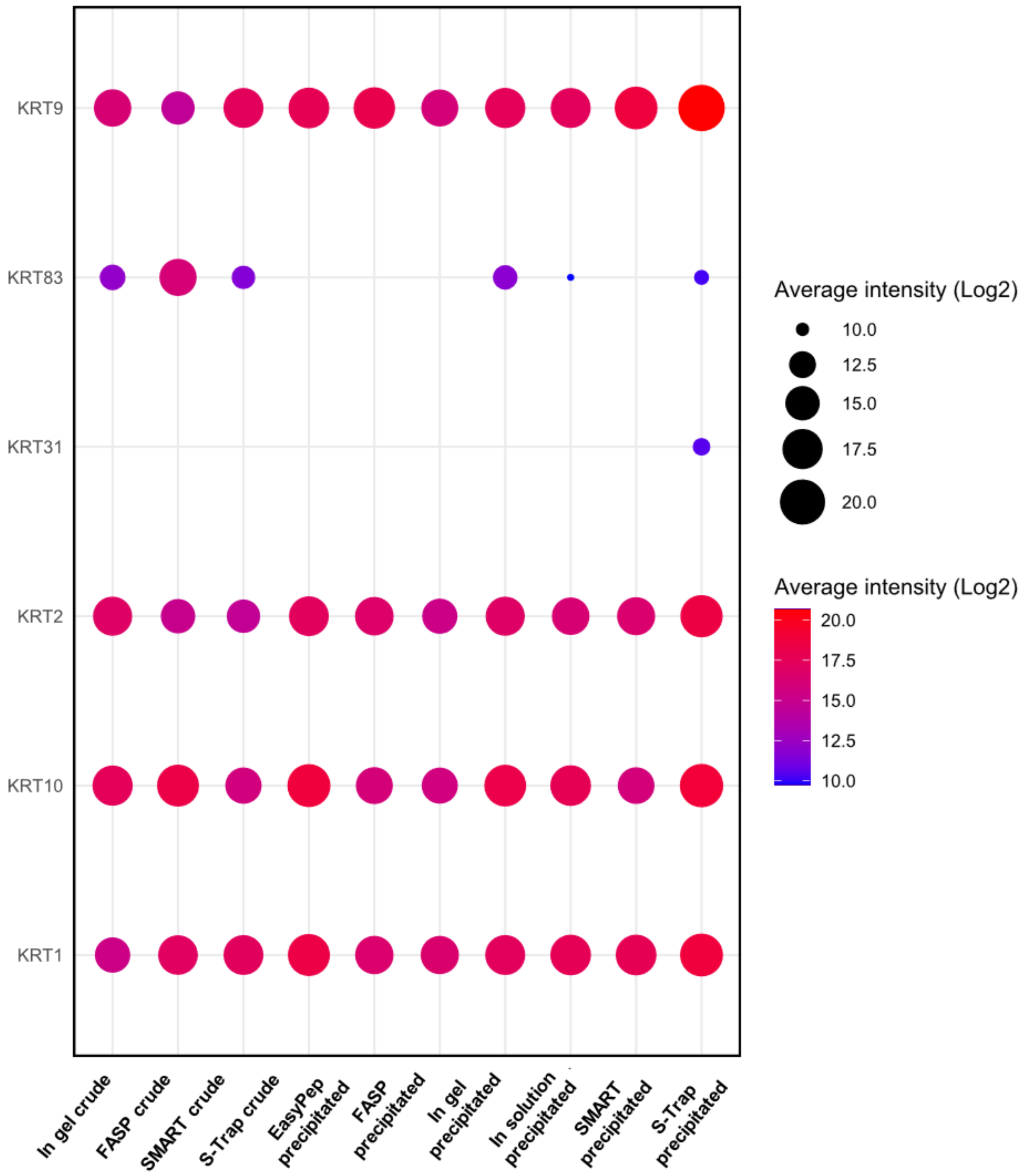

Supplementary figure 3. Presence of potential contaminant proteins. Dot plot showing intensity (dot size and colour) of potential human contaminant proteins identified in proteomics analysis of bile.

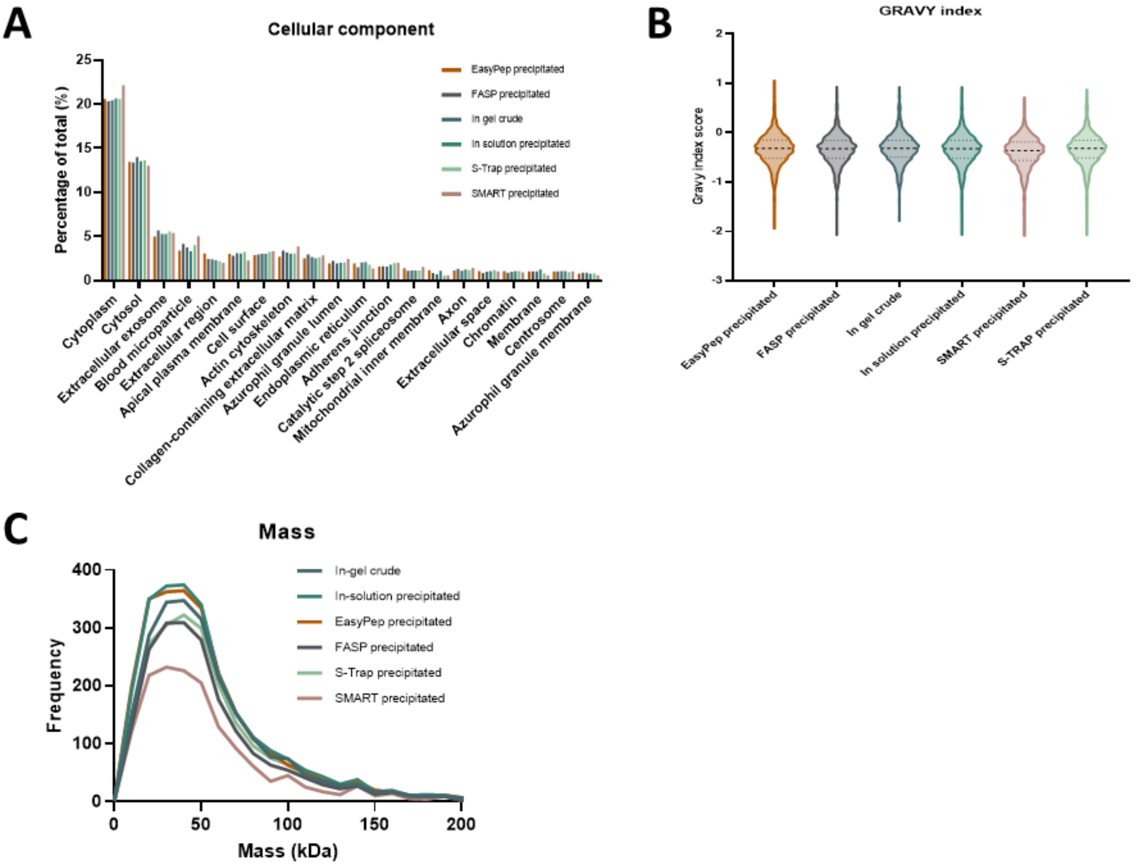

Supplementary figure 4. Protein characteristics in the top 6 selected methods. (A) Bar chart showing top 20 most frequent Gene Ontology cellular components. (B) Grand average of hydropathicity index (GRAVY) scores for proteins identified in each method. (C) Plot showing frequency of protein masses in each method.

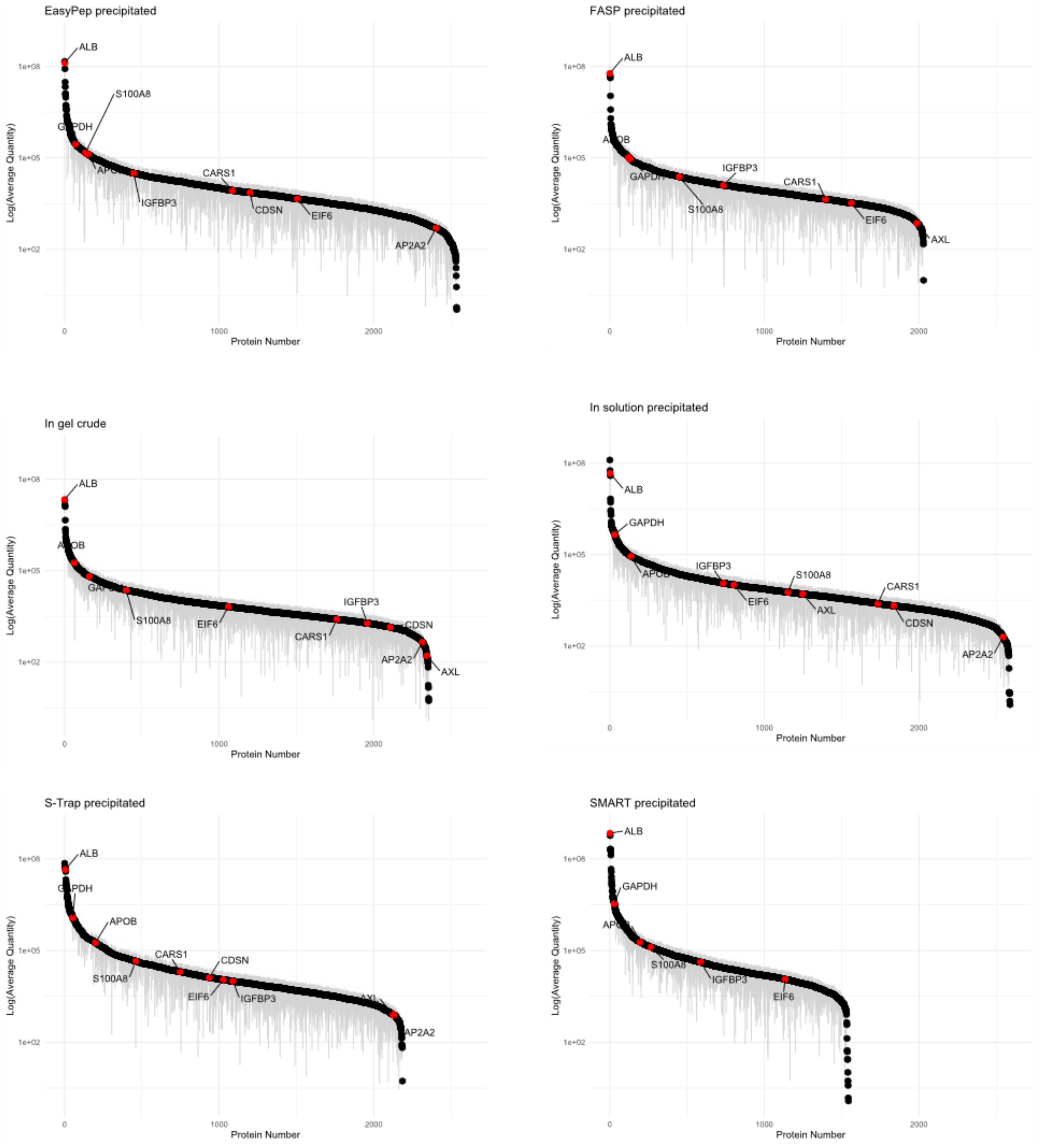

Supplementary figure 5. Protein intensity dynamic ranges in the top 6 selected methods. Dynamic range plots showing intensity for all proteins identified in each to the top 6 selected methods. Ten highlighted proteins (red) show variation in intensity across methods.

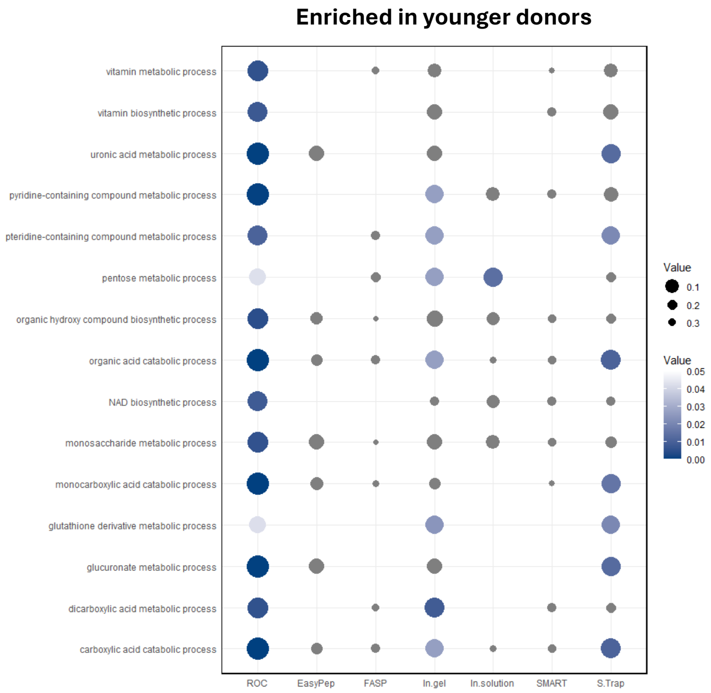

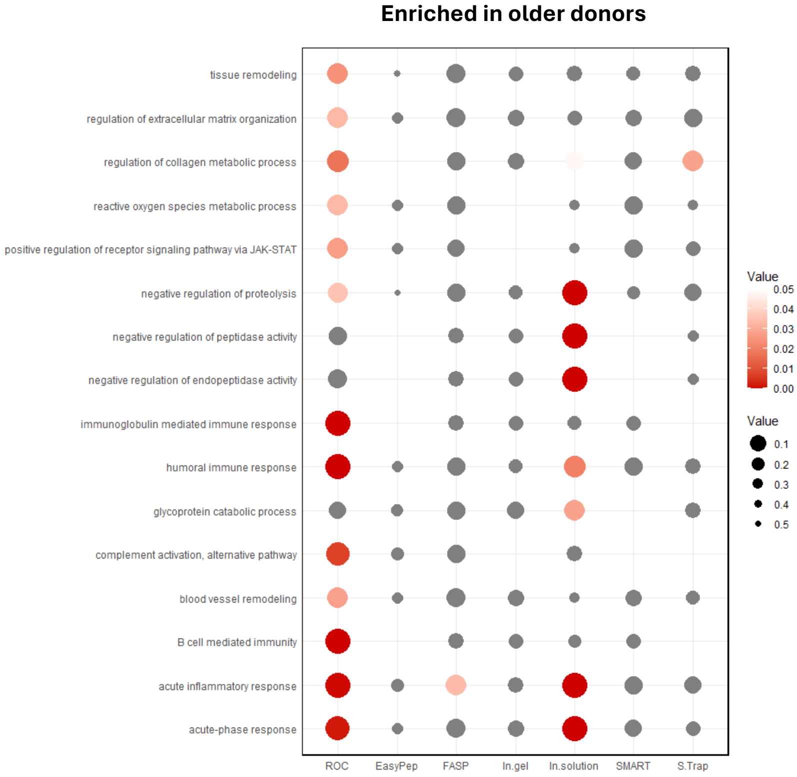

Supplementary figure 6. Biological comparison between younger and older donors. Dot plots showing larger selection of identified Gene Ontology biological processes in younger and older donors. Color gradients represent adjusted p-value of significant biological processes. Grey is used for processes where involved proteins are present, but the process did not reach significance. The size of each point represents adjusted p-value in significant and non-significant biological processes.
